# Gemistocytic Differentiation in Isocitrate Dehydrogenase Mutant Astrocytomas: A Histopathological and Survival Analysis

**DOI:** 10.7759/cureus.37542

**Published:** 2023-04-13

**Authors:** Usman Hassan, Faizan Amer, Mudassar Hussain, Sajid Mushtaq, Asif Loya, Muhammad Abu Bakar

**Affiliations:** 1 Pathology, Shaukat Khanum Memorial Cancer Hospital and Research Centre, Lahore, PAK; 2 Biostatistics and Epidemiology, Shaukat Khanum Memorial Cancer Hospital and Research Centre, Lahore, PAK

**Keywords:** idh mutation, survival, prognosis, astrocytoma, gemistocytes

## Abstract

Gemistocytic differentiation is a rare histological feature seen in IDH mutant Astrocytomas. The 2021 World Health Organization (WHO) retains the diagnosis of IDH mutant Astrocytoma with its classical histology and tumors with the rare histological pattern of g*emistocytic* differentiation. *Gemistocytic* differentiation has historically been associated with a worse prognosis and shorter survival, and this prognostic difference has not been investigated in detail in our population.

A population-based retrospective study included 56 patients with IDH mutant Astrocytoma with Gemistocytic differentiation and IDH mutant Astrocytoma diagnosed between 2010 and 2018 in our hospital. Demographic, histopathological, and clinical parameters were compared between the two groups. Gemistocyte percentage, perivascular lymphoid infiltrates, and Ki-67 proliferation index were also analyzed. A Kaplan-Meier analysis was done to analyze any prognostic difference in the overall survival time between the two groups.

Patients with an IDH mutant Astrocytoma having g*emistocytic* differentiation had an average survival period of 2 years, while patients diagnosed with an IDH mutant Astrocytoma had an average survival time of approximately 6 years. There was a statistically significant decrease in survival time (p = 0.005) for patients with tumors with g*emistocytic* differentiation. The percentage of gemistocytes and the presence of perivascular lymphoid aggregates did not correlate with survival time (p = 0.303 and 0.602, respectively). Tumors with g*emistocytic* morphology had a higher mean Ki-67 proliferation index (4.4%) than IDH mutant Astrocytoma (2.0%, p = 0.005).

Our data suggest that IDH mutant Astrocytoma with Gemistocytic differentiation is an aggressive variant of IDH mutant Astrocytoma associated with a shorter survival time and an overall worse prognosis. This data might be helpful to clinicians in the future management of IDH mutant Astrocytoma with Gesmistocytic differentiation as an aggressive tumor.

## Introduction

The classification of Glial Neoplasms has undergone extensive changes in the past few years. The 2021 World Health Organization (WHO) classification of Central Nervous System (CNS) neoplasms has changed from a purely phenotypical classification to one that also incorporates genotypic subtyping of glial neoplasms to provide a more stratified system of classifying CNS neoplasms [1]. Particular emphasis is made on Isocitrate Dehydrogenase (IDH) mutations in glial neoplasms and the associated better prognosis [2].

IDH mutant Astrocytoma is an infiltrative glioma of neoplastic astrocytes that diffusely invade glial tissue. Its diagnosis is supported by mutations of p53 and ATRX genes [3,4]. IDH mutation analysis is now a prerequisite for diagnosing an Astrocytoma, and research shows that these mutations appear to confer a significant survival advantage over their wild-type counterparts [5-9]. IDH is a Tricarboxylic Acid Cycle enzyme that converts Isocitrate into α-ketoglutarate (α-KG) [10]. It has three isoenzymes encoded by five genes, with IDH-1 and IDH-2 involved in developing human Gliomas [10]. IDH 1 and IDH 2 usually are responsible for producing nicotinamide adenine dinucleotide phosphate (NADPH). IDH-1 mutations are the most common and almost always result from a missense amino acid substitution of arginine to histidine [10]. This mutation causes excessive oncometabolite 2-hydroxyglutarate and decreased NADPH production [11,12]. This oncometabolite has numerous consequences, including DNA hypermethylation in gene promoter regions, silencing of expression of several important cellular differentiation factors, and increased susceptibility to oxidative stress [13,14]. Why these mutations are associated with a better survival outcome is not entirely understood. However, studies show that IDH mutations may alter the immune response against the tumor, resulting in overall less aggressiveness [15]. The grading of these tumors is based on the degree of atypia, presence of necrosis, and vascular proliferation, and more recently, based on molecular analysis for CDKN2A and CDKN2B mutations. Ki-67 proliferation index is usually low for Grade 2 tumors.

IDH mutant Astrocytoma with Gemistocytic Differentiation (abbreviated as IDH mutant GA for this study) is a rare histological variant of IDH mutant Astrocytoma. It is now the only variant recognized by the WHO CNS classification. These diffusely infiltrative gliomas are characterized by large cells having voluminous, eosinophilic/glassy cytoplasm, an eccentrically placed nucleus, and fine, branching cytoplasmic processes [16,17]. According to the 2021 WHO classification, at least 20% of the cell population of an astrocytic neoplasm must consist of gemistocytes to be labeled as a dominant tissue pattern. The cellularity of these tumors is usually greater than IDH mutant Astrocytomas. Another commonly identified histological is perivascular lymphoid infiltrates, which appear as concentrically arranged lymphoid collections around blood vessels. IDH mutant GA has graded similarly to IDH mutant Astrocytoma. This tissue pattern may be associated with a focal gain of chromosome 12p encompassing CCND2 [18].

Even though IDH mutant tumors are generally associated with a favorable prognosis, few studies show that the IDH mutant GA may be associated with a worse clinical outcome and shorter survival than IDH mutant Astrocytomas, even when the tumor grades are the same [19,20]. Further studies are required to establish a definite association, and no such studies have been conducted regarding this in the Pakistani population.

## Materials and methods

This study was conducted in the pathology department of Shaukat Khanum Memorial Cancer Hospital and Research Center, Lahore, Pakistan. The study was first submitted to the hospital's Internal Review Board for approval. A waiver of informed consent (IRB number EX-25-11-20-01) was obtained since no intervention would be done on the participants. The clinical data were obtained from hospital records for the in-house patients and via telephonic conversations for review cases. Clinical data included parameters such as current status (alive or dead), date of death, and history of therapy (both chemotherapy and radiotherapy were included in the study). The survival period began from the date of diagnosis by a biopsy until the patient's death due to the disease. Patients who died from unrelated causes were not included in the study. The exact details of the nature of the therapy, its duration, and its dosage were not available to us.

The search engine of the Hospital Information System was used to retrieve cases of IDH mutant GA and IDH mutant Astrocytoma diagnosed between 2010 and 2018. The slides of all cases (including Hematoxylin, Eosin, and immunohistochemistry) were retrieved from the hospital archive and reviewed by two neuropathologists. Any discrepancies were removed by discussing them with the primary pathologist. For IDH mutant GA, an estimate of gemistocyte percentage was noted in each case and was tiered between 20-60% and more than 60%. The presence or absence of perivascular lymphoid aggregates was also noted in each case. All selected cases were graded as Grade 2 based on their morphology to maintain standardization. IDH immunohistochemistry was unavailable to us before 2016, so IDH immunostaining was performed on all cases diagnosed before 2016. The IDH immunostaining available in our department is the Bio-SB IDH 1 antibody (clone: IHC 132). It was performed using a Dako-Link 48 Auto Stainer and a Bond Polymer Refine Detection System. Immunostaining for IDH-2 is unavailable in our department, and a PCR to confirm a negative IDH-1 mutation status could not be performed. Ki-67 proliferation index was also noted in both groups. Molecular analysis for CDKN2A/CDKN2B or CCND2 mutations is not available in our laboratory.

Cases with classical histological features, satisfactory results of IDH immunohistochemistry, and adequate clinical data were included in the study. All cases of IDH mutant Astrocytoma had a loss of ATRX expression and aberrant p53 expression. Cases with inconclusive histological features on review, equivocal or negative results with IDH immunohistochemistry, or with no follow-up available were not selected. Sixty-two cases of Astrocytomas with Gemistocytic differentiation were reviewed. No follow-up was available for 26 cases; thus, they were excluded from the study. Four cases were excluded due to inconclusive histological features, and two were excluded due to insufficient material on the blocks. IDH immunostaining was done on 14 cases; two showed negative results. They were excluded from the study, leaving 28 cases for subsequent analysis. In the comparison group, 65 cases of astrocytomas were reviewed. No follow-up was available for 31 cases, because of which they were excluded from the study. IDH immunostaining was done on 16 cases, and six showed negative results. They were excluded from the study, leaving 28 cases for subsequent analysis.

The statistical analysis was performed on IBM SPSS Software Version 26. Continuous variables were reported as mean and standard deviation, while categorical variables were presented as frequency and percentages. The chi-square test bifurcated the categorical variables concerning the outcome variable (current status). The independent t-test was used to check the mean difference of continuous variables concerning the outcome variable. Overall survival (disease-specific survival) analysis was done to check the survival differences between the two groups. The Kaplan-Meier curve was drawn to plot the survival difference, and the log-rank test compared Kaplan-Meier plots. p-values less than 0.05 were considered statistically significant.

## Results

Demographics

Twenty-eight cases of IDH mutant GA and IDH mutant Astrocytoma fulfilled the inclusion criteria. The demographic parameters are summarized in Table 1.

**Table 1:** Demographics IDH-Mutant GA: IDH mutant Astrocytoma with Gemistocytic Differentiation

	IDH-Mutant GA	IDH-Mutant Astrocytoma
Frequency (n)	28	28
Gender (n)		
Male	20	16
Female	8	12
Age (years)		
Mean	35.5	34.9
Standard Deviation	11.4	12.5
Range	16-58	10-60
Location, n (%)		
Frontal Lobe	11 (39.3)	15 (53.6)
Parietal Lobe	11 (39.3)	06 (21.4)
Occipital Lobe	05 (17.9)	04 (14.3)
Temporal Lobe	01 (03.6)	01 (03.6)
Thalamus	00	02 (07.1)

Histopathological features

**Figure 1:**
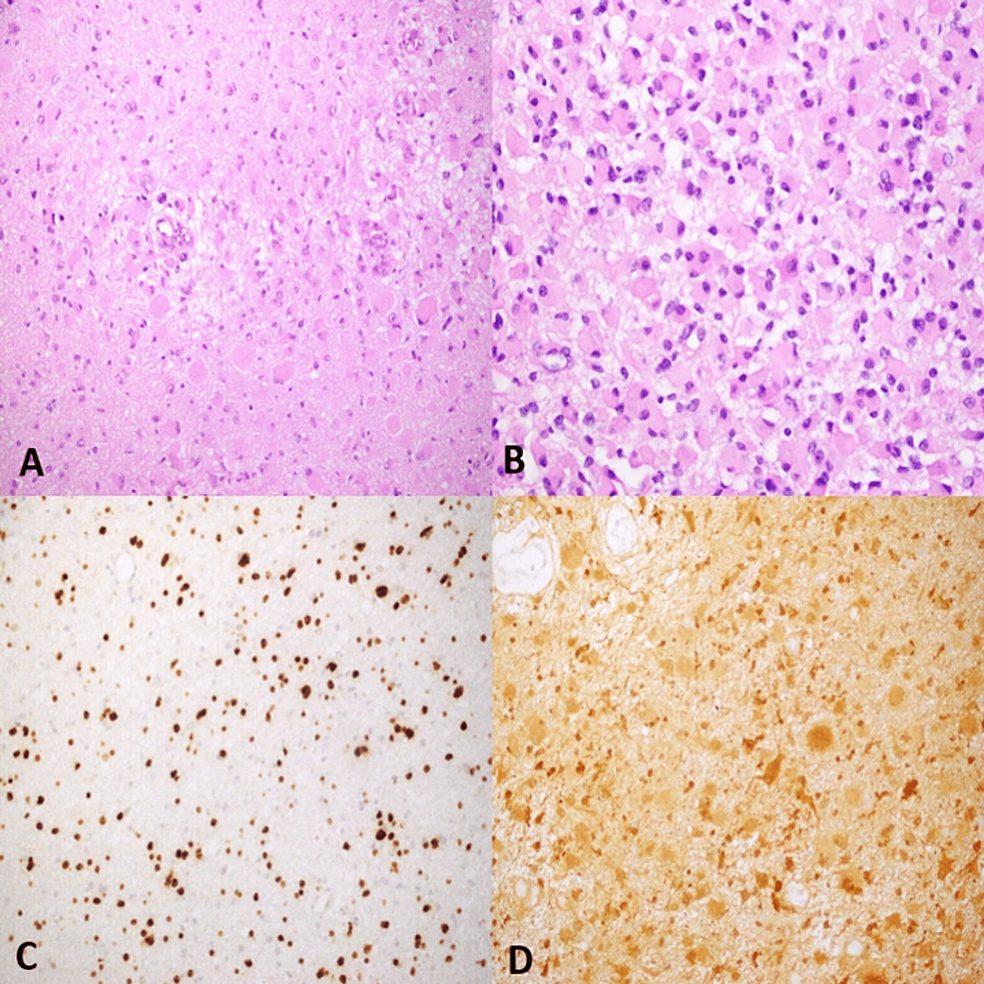
IDH mutant Astrocytoma with Gemistocytic Differentiation (A&B) The tumor comprises sheets of large, plump cells with abundant eosinophilic cytoplasm and eccentric nuclei. Tumor cells are scattered in a background of smaller glial cells (20x). (C) Tumor cells showing strong nuclear reactivity for the glial marker Olig-2. (D) Diffuse cytoplasmic expression of Isocitrate Dehydrogenase-1 (IDH-1).

The most characteristic histological feature of IDH mutant GA in our study was sheets of gemistocytes with intermixed neoplastic astrocytes in a neurofibrillary background. The gemistocytes showed eccentric, hyperchromatic nuclei and abundant, brightly eosinophilic cytoplasm (Figure 1). These cells showed mild to moderate cytological atypia. The background neurofibrillary matrix showed occasional inflammatory cells and foci of hemorrhage and infarction. Perivascular lymphoid infiltrates were also identified (n = 10, 34.7%). The percentage of gemistocytes was estimated as being greater than 60% (pure gemistocytic morphology, n = 20, 71.4%) and between 20-60% (mixed morphology, n = 8, 28.6%). Only a rare mitotic figure was identified in selected cases. Since all cases were grade 2 tumors, we did not see necrosis, vascular proliferation, or unusual/anaplastic features. The mean Ki-67% was 4.4% (range = 1-9%) in the background neoplastic cells, and the gemistocytes did not show labeling with the Ki-67 antibody. All tumors included in our study showed strong cytoplasmic expression of IDH-1.

**Figure 2:**
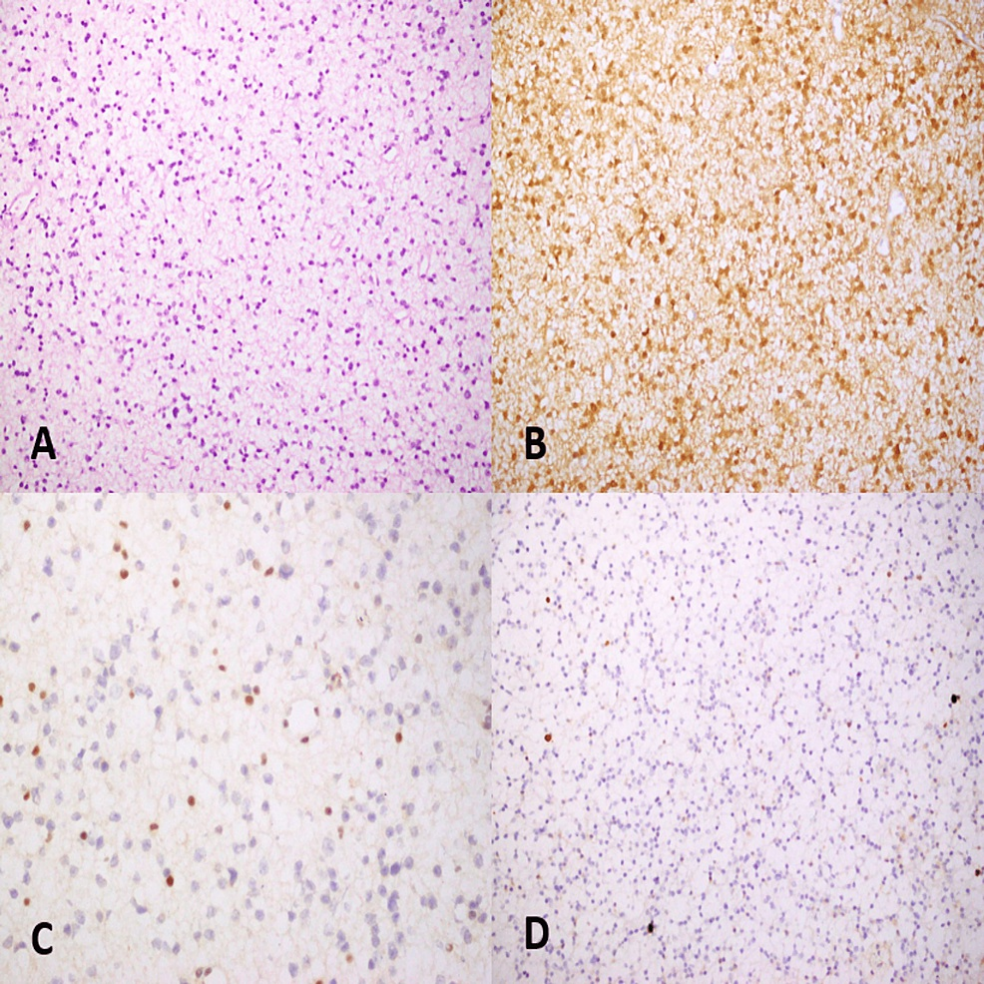
IDH mutant Astrocytoma (A) The tumor is characterized by well-differentiated, fibrillary glial cells that diffusely invade non-neoplastic glial tissue. The cellularity is low, and there is mild cellular atypia (20x). (B) Isocitrate Dehydrogenase-1 (IDH-1) positivity is characterized by strong cytoplasmic expression. (C) There is a characteristic loss of ATRX expression with positive internal control in non-neoplastic astrocytes. (D) This tumor had a Ki-67 proliferation index of 1%.

In the IDH mutant Astrocytoma comparison group, the tumors were characterized by diffuse sheets of astrocytes infiltrating into the surrounding non-neoplastic glial tissue. The neoplastic astrocytes had indistinct cell boundaries, mild pleomorphism, and hyperchromatic nuclei (Figure 2). The cellularity of these tumors was predominantly low in most cases. The background tissue is comprised of neurofibrils and non-neoplastic glial cells. The mitotic count was extremely low, and the mean Ki-67 proliferation index was 2% (range = 1-5%). IDH-1 was strongly positive in all the cases included in our study.

Prognostic analysis

**Figure 3:**
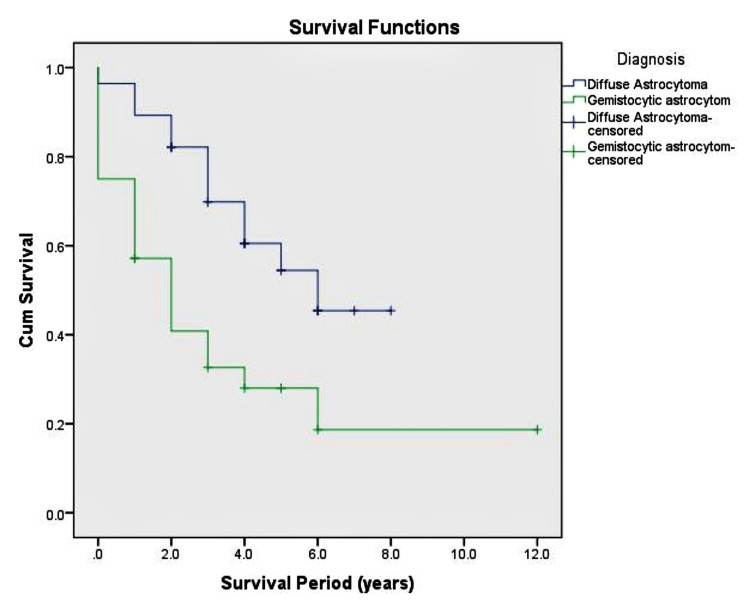
Kaplan Meier Survival Analysis The disease-specific survival difference between IDH mutant GA and IDH mutant Astrocytomas was analyzed to assess the prognostic difference. The IDH mutant GA group patients had a significantly decreased survival time compared to the IDH mutant Astrocytoma group.

In the IDH mutant GA group, 8 (28.4%) patients were alive at the time of the study's commencement, while 20 (71.4%) patients had passed away due to disease-related complications. In the comparison group, 16 (57.1%) patients were alive at the time of the study's commencement, and 12 (42.9%) patients had passed away due to their disease (p = 0.029). A statistically significant overall survival difference was observed between patients with IDH mutant GA and IDH mutant Astrocytoma. The mean overall survival time for all patients in both groups was approximately 4 years. Additionally, the median overall survival times of IDH mutant Astrocytoma and IDH mutant GA were 6 and 2 years, respectively (p = 0.005). The survival times are illustrated in Figure 3.

**Figure 4:**
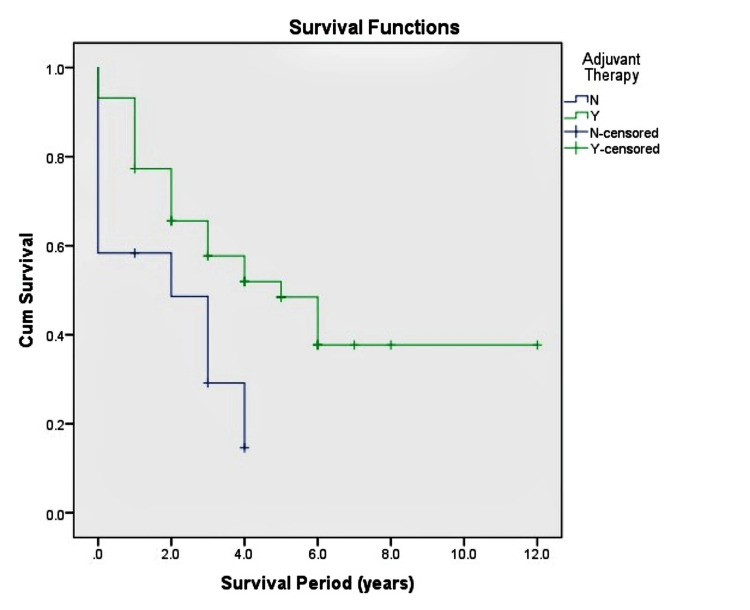
Adjuvant Therapy Adjuvant Therapy was associated with an improvement in the survival period in both groups.

Nineteen patients (67.9%) with IDH mutant GA and 25 cases (89.3%) of IDH mutant Astrocytoma reported a history of adjuvant therapy for their disease. As expected, adjuvant therapy was strongly associated with a significant increase in overall survival time (p = 0.022), with patients who had received therapy being alive twice as long as patients who did not receive adjuvant therapy (Figure 4). However, we did not find an association between the histological diagnosis and the prognostic impact of adjuvant therapy since neither group showed a significant increase in survival time compared to the other. Since we have yet to have the exact details on the type and duration of therapy given, we cannot conclusively report whether adjuvant therapy causes a statistically significant increase in survival time.

**Figure 5:**
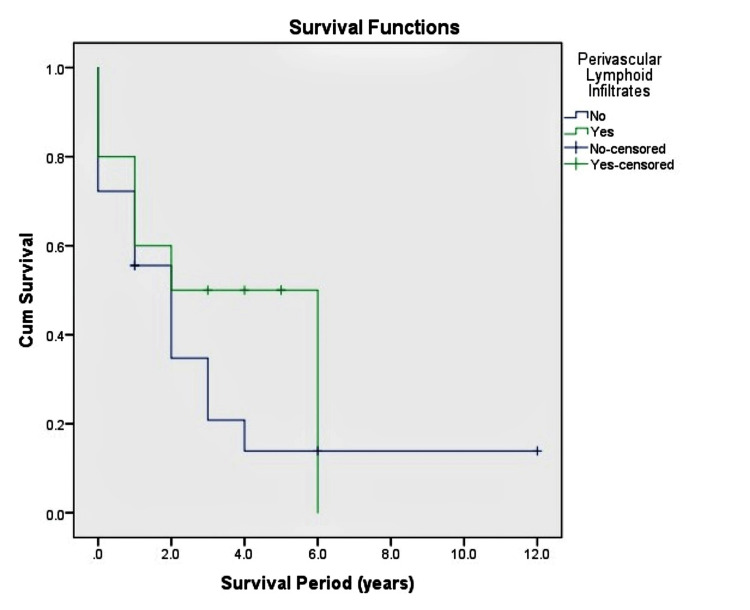
Disease-specific survival to perivascular lymphoid infiltrates. The findings do not show a statistically significant correlation between survival and the presence of perivascular lymphoid infiltrates.

**Figure 6:**
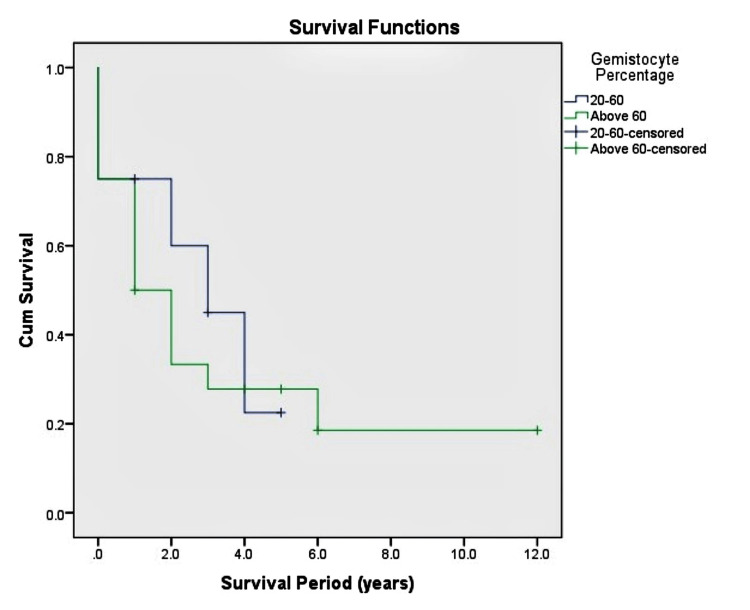
Disease-specific survival to Gemistocyte percentage. There was no correlation between survival and the percentage of gemistocytes.

Within the IDH mutant GA group, perivascular lymphoid aggregates or the gemistocyte percentage did not correlate with survival time (p = 0.303 and p = 0.602, respectively), as highlighted in Figures 5, 6 respectively.

## Discussion

This study is a retrospective analysis of IDH mutant GA compared to IDH mutant Astrocytomas in the Pakistani population. The WHO Central Nervous System classification has significantly changed in recent years. The 2021 classification now divides adult-type diffusely infiltrative gliomas into IDH mutant Astrocytomas, Oligodendrogliomas, and IDH wild-type Glioblastomas [1]. Recent advances in molecular techniques have identified several mutations with a profound prognostic impact, including Isocitrate Dehydrogenase (IDH) [21]. The IDH mutant Astrocytomas are a commonly encountered group of tumors encompassing low-grade infiltrative astrocytomas to high-grade tumors with histological overlap with Glioblastomas. These IDH mutant tumors have been recently categorized separately from their Wild-type counterparts, as they have a much better prognosis. IDH mutation analysis is now mandatory for their diagnosis. The IDH mutant Astrocytoma with Gemistocytic Differentiation (IDH mutant GA) is a rare histological subtype of IDH mutant Astrocytoma. It is the only subtype that is a part of the WHO CNS tumor classification. It was first described in the early 20th century, with its name being derived from the Greek word 'gemistos' (filled/stuffed), and it denotes the neoplasm's most prominent cell type, the Gemistocyte. The gemistocytes are large cells with abundant, eosinophilic, glassy cytoplasm and peripherally oriented, hyperchromatic nuclei. Gemistocytes can also be identified in other gliomas, where they are proposed to be reactive cells; however, Kros et al. [17] and Reis et al. [22] proved them to be neoplastic cells in their studies. IDH mutant GA are graded in the same way as IDH mutant Astrocytomas, however tumors of the same grade as IDH mutant Astrocytomas have been associated with an aggressive course, thus highlighting the importance of recognizing this histological and the possible need for more aggressive medical management.

Our study found that IDH mutant GA are usually supratentorial and show a predilection for the frontal and parietal lobes of the cerebral cortex. In comparison, most IDH mutant Astrocytomas are found in the frontal lobes. Simkin et al. [23] describe rare Astrocytomas with Gemistocytic differentiation occurring within the brainstem and spinal cord, but we did not encounter any such case in our study. The IDH mutant GA showed a Male to Female ratio of 2.5 to 1 in contrast to the 1.3:1 for IDH mutant Astrocytomas.

Our study's mean age for patients with IDH mutant GA was 35.5 years. Avinder et al. [24] reported a similar age distribution within the Indian population, and Martins et al. [25] also reported a similar age distribution for IDH mutant GA. However, Okamoto et al. [26] reported a significantly different age distribution in their study, with a mean age of 50 years in the Swiss population. In the comparison group, the mean age for IDH mutant Astrocytomas in our study was 34.9 years, significantly lower than that reported in the literature (mean 45 years) [26-28]. Moreover, we did not observe any significant difference in the mean age of disease presentation between the two groups (p=0.549). These findings highlight that IDH mutant GA occurs within the same age distribution as IDH mutant Astrocytomas in the South Asian population.

Our results showed that with tumor grade being a constant factor and with overall similar age distribution, the mean overall survival period of IDH mutant GA is 2 years. This was shorter than the mean survival time reported by Okamoto et al. [26], which is 3.2 years. The mean overall survival time for IDH mutant Astrocytoma was significantly higher (6 years, p=0.005) than IDH mutant GA. Okamoto et al. [26] reported a mean survival time of 7 years for Astrocytomas in their study. These results show that the overall survival period in both tumor groups is lower in our population than in others, even though these tumors have been shown to occur in a younger age group in our population. These differences in survival in our population are likely due to multiple factors. Patients in our region tend to present late to the physician for their clinical symptoms, delaying the diagnosis of these tumors. Limited treatment options are available for these tumors, and many are too expensive for most of the population. Clinical follow-up and compliance are also generally poor in our region.

70% (n=20) of IDH mutant GA had a Gemistocyte percentage of greater than 60%, and 36% of the cases had Perivascular Lymphoid Aggregates. Neither of these features correlated with survival time in our study (p=0.303 and p=0.602, respectively). These results were similar to the ones reported by Krouwer et al. [29] and Yang et al. [30]. Evaluating Gemistocyte percentage and perivascular lymphoid infiltrates may be more of academic interest. Several studies, including ours, highlight that neither of these parameters correlates with survival time in IDH mutant GA.

The average Ki-67 proliferation index for IDH mutant GA in our study was 4.4% which was higher than the one reported by Avinder et al. [24], Martins et al. [25], and Yang et al. [30], who reported mean Ki-67 as 3.7%, 3.3%, and 2.5% respectively. This Ki-67 proliferation index was also significantly higher compared to the comparison group of IDH mutant Astrocytomas (2.0%, p=0.005). Despite both groups having tumors graded as 2 on morphology, IDH mutant GA consistently showed a higher Ki-67 proliferation index. This higher Ki-67 index, despite a grade 2 morphology, may indicate the inherent aggressiveness of this tumor. This finding also raises the possibility of including the Ki-67 proliferation index as a part of the grading criteria for the IDH mutant GA. This may allow better grade-to-prognosis correlation for these tumors and may allow clinicians to make more informed decisions in the treatment of these tumors.

## Conclusions

This is a single institution-based retrospective study on a rare variant of central nervous system Astrocytomas. To our knowledge, no other study has been conducted on this in our population. Limitations include the small number of cases available for evaluation in our hospital and the unavailability of IDH-2 immunohistochemistry and PCR techniques to allow the inclusion of more cases in the study. Despite the limitations in our methodology, our study contributes to the hypothesis that IDH mutant Astrocytoma with Gemistocytic differentiation (IDH mutant GA) is an aggressive histological variant of IDH mutant Astrocytoma, is associated with an overall decreased survival time, and should be managed more aggressively. We have also shown that the percentage of gemistocytes within an IDH mutant GA and the presence of perivascular lymphoid aggregates do not correlate with survival time or a worse prognosis.
